# The Effectiveness of Facial Expression Recognition in Detecting Emotional Responses to Sound Interventions in Older Adults With Dementia

**DOI:** 10.3389/fpsyg.2021.707809

**Published:** 2021-08-25

**Authors:** Ying Liu, Zixuan Wang, Ge Yu

**Affiliations:** Key Laboratory of Cold Region Urban and Rural Human Settlement Environment Science and Technology, Ministry of Industry and Information Technology, School of Architecture, Harbin Institute of Technology, Harbin, China

**Keywords:** facial expression recognition, sound intervention, emotion, type of sound source, elderly with dementia

## Abstract

This research uses facial expression recognition software (FaceReader) to explore the influence of different sound interventions on the emotions of older people with dementia. The field experiment was carried out in the public activity space of an older adult care facility. Three intervention sound sources were used, namely, music, stream, and birdsong. Data collected through the Self-Assessment Manikin Scale (SAM) were compared with facial expression recognition (FER) data. FaceReader identified differences in the emotional responses of older people with dementia to different sound interventions and revealed changes in facial expressions over time. The facial expression of the participants had significantly higher valence for all three sound interventions than in the intervention without sound (p < 0.01). The indices of sadness, fear, and disgust differed significantly between the different sound interventions. For example, before the start of the birdsong intervention, the disgust index initially increased by 0.06 from 0 s to about 20 s, followed by a linear downward trend, with an average reduction of 0.03 per 20 s. In addition, valence and arousal were significantly lower when the sound intervention began before, rather than concurrently with, the start of the activity (*p* < 0.01). Moreover, in the birdsong and stream interventions, there were significant differences between intervention days (*p* < 0.05 or *p* < 0.01). Furthermore, facial expression valence significantly differed by age and gender. Finally, a comparison of the SAM and FER results showed that, in the music intervention, the valence in the first 80 s helps to predict dominance (*r* = 0.600) and acoustic comfort (*r* = 0.545); in the stream sound intervention, the first 40 s helps to predict pleasure (*r* = 0.770) and acoustic comfort (*r* = 0.766); for the birdsong intervention, the first 20 s helps to predict dominance (*r* = 0.824) and arousal (*r* = 0.891).

## Introduction

Dementia is a set of syndromes characterized by memory and cognitive impairment caused by brain diseases. China currently has the largest population of older people with dementiain the world, ~14 million (Jia et al., 2020). The decline in cognitive function causes older adults with dementia to gradually lose the ability and opportunities to engage in various activities, and scarce activity can easily induce depression and agitation behavior (Mohler et al., 2018). Lack of external stimuli is a prominent cause of negative emotions in older people with dementia. Studies have found that sensory stimulation through acoustic intervention can reduce the agitation behavior of older people with dementia (Riley-Doucet and Dunn, 2013; Nishiura et al., 2018; Syed et al., 2020). Therefore, how to create a healthy acoustic environment for the older with dementia has become an urgent problem for countries worldwide to be solved.

Emotions can be perceived and evaluated as their status changes with change in the person–environment relationship (Rolls, 2019). Despite their cognitive impairment, older adults with dementia continue to display emotions, and their internal emotional processing may be intact or partially retained (Satler et al., 2010); specifically, they retain the feeling and acquisition of emotions (Blessing et al., 2006). In addition, the emotions reflected by facial expressions are similar between older adults with mild dementia and typical older adults (Smith, 1995). Along with the decline in cognitive function, older adults with dementia can experience various emotional problems, such as anxiety, depression, and excitement. At present, there are no effective treatment methods and drugs for dementia. Therefore, effective emotional intervention is especially important to suppress negative emotions and generate positive emotions (Marquardt et al., 2014). Common methods include environmental intervention, behavioral intervention, psychological intervention, social therapy, and entertainment therapy (Howe, 2014).

Some previous studies have shown that environmental interventions can play a therapeutic role for older adults with dementia (Satariano, 2006). In this regard, the acoustic environment is important, and appropriate sound interventions can help delay the onset of dementia (Wong et al., 2014). Music has been widely used in treating dementia during the past decade, and remarkable results have been achieved with respect to memory and mood disorders (Ailun and Zhemin, 2018; Fraile et al., 2019). Music can reduce depression (Li et al., 2019) and improve behavioral disorders, anxiety, and restlessness in older people with dementia (Gomez-Romero et al., 2017). In addition, some studies have investigated how best to design the acoustic environment for older adults with dementia based on the phenomenon of auditory masking (Hong et al., 2020). For example, adding white noise to the environment may mitigate some auditory hallucinations, helping older adults with dementia to temporarily relax. White noise can also reduce the mental and behavioral symptoms of older adults with dementia (Kaneko et al., 2013). Conversely, some studies have revealed a negative impact of noise on the quality of life of older people with dementia. For example, some studies showed that high noise can lower the social mood of elderly with dementia and induce falling behaviors (Garre-Olmo et al., 2012; Jensen and Padilla, 2017). When the daytime noise level is continuously higher than 55 dBA, it can induce emotional and behavioral agitation in older adults with dementia (Harding et al., 2013). The current development trend of the acoustic environment is changing from noise control to soundscape creation, that is, from reducing negative health effects to promoting positive health trends (Kang et al., 2020). However, research on how the acoustic environment can promote the health of older adults with dementia has so far only focused on music and noise. Whether other types of sound interventions, such as birdsong and stream sound, improve mood and health in older people with dementia have not been examined. In addition, some studies have proved that the playing time of a sound source is also an important factor affecting the perception of sound in people (Staats and Hartig, 2004; Korpela et al., 2008). However, there is no relevant research on whether the time of intervention of the sound source will affect emotions.

Prior research on emotions has mainly been conducted at the three levels, namely, physiology, cognition, and behavior. Different research levels correspond to different research contents and methods (Zhaolan, 2005). However, wearing physiological measuring devices may induce negative emotions in older people with dementia, who are more prone to mood swings.

Most common in emotion research is the study of cognitive theory, which posits that a stimulus can only produce a specific emotion after the cognitive response of the subject (Danling, 2001). The main method adopted is a subjective questionnaire. For example, Meng et al. (2020b) studied the influence of music on communication emotions through a field questionnaire, which asked participants to evaluate their emotional state. Zhihui (2015) and Xie et al. (2020) conducted field experiments in train stations and hospital nursing units, asking participants how various types of sound sources affect their emotions. However, surveys have several limitations. First, the questionnaire is subjective, and an “experimenter effect” might occur if the questionnaire is not well-designed (Brown et al., 2011). Second, a single-wave survey cannot show trends over time in how participants react to a sound intervention, which precludes calculating the role of time in the intervention process.

The third main research avenue is the study of behavioral emotions. Behaviorists believe that external behaviors caused by emotions can reflect the true inner feeling of a person (Yanna, 2014). The main method is to measure emotional changes in people through facial, verbal, and bodily expressions. Psychologists generally believe that expressions are a quantitative form of changes in emotions. As a tool for evaluating emotions, the software FaceReader, based on facial expression recognition (FER), has been applied in psychological evaluation (Bartlett et al., 2005; Amor et al., 2014; Zarbakhsh and Demirel, 2018). The effectiveness of FER has been proven in many previous studies, and it can measure emotions with more than 87% efficacy (Terzis et al., 2010). The validity of FaceReader for East Asian people, in particular, has been shown to be 71% (Axelsson et al., 2010; Yang and Hongding, 2015). The efficiency of this method has been tested in many research fields. For example, Hadinejad et al. (2019) proved that, when participants watched travel advertisements, arousal and positive emotions diminished. Leitch et al. (2015) found that the length of time after tasting sweeteners affected the potency and arousal of facial expressions. In addition, Meng et al. (2020a) conducted laboratory experiments to test the effectiveness of facial expressions for detecting sound perception and reported that the type of sound source had a significant impact on the valence and indicators of facial expressions. FER has also been used in research on the health of older adults with dementia. Re (2003) used a facial expression system to analyze the facial expression patterns and facial movement patterns of older people with severe dementia. Lints-Martindale et al. (2007) measured the degree of pain of older adults with dementia through a facial expression system. However, no study has tested whether FER can be used to investigate the effect of the acoustic environment on the emotions of elderly with dementia. In addition, the characteristics of the normal population, such as their gender, age, and so on, are related to their emotions (Ma et al., 2017; Yi and Kang, 2019). However, in older adults with dementia, it is not clear whether these characteristics affect the results of facial expression.

To address this gap in the literature, this study explored the effectiveness of FER in measuring how sound interventions affect the emotions of elderly with dementia. Specifically, this study is focused on the following research questions: (1) Can facial expression analysis systems be used to study sound interventions on the emotions of older people with dementia?; (2) How do different types of sound interventions affect the valence and other indicators of facial expressions of elderly with dementia?; (3) Do demographic and time factors, such as age, gender, Mini-Mental State Examination (MMSE) scores, intervention duration, and intervention days, cause different degrees of impact? A field experiment was conducted to collect facial expression data of 35 elderly with dementia in an older adult care facility in Changchun, China. The experiment included three sound sources typically preferred by elderly with dementia: music, stream, and birdsong.

## Materials and Methods

### Participants

The participants in this study are older people with dementia residing at seven institutes in Changchun, China. A total of 35 older people with mild dementia was selected, comprising 16 men and 19 women aged 60–90 years (mean = 81, *SD* = 7). The number of participants was determined based on similar related experiments (El Haj et al., 2015; Cuddy et al., 2017).

The following selection criteria were applied. First, participants had to be at least 60 years old. Second, participants had to score 21–27 on the MMSE, indicating mild cognitive impairment or dementia. Third, participants had to be able to communicate through normal conversation and have normal hearing. Fourth, participants were required to have <5 years of music training to ensure that the music intervention induced cognitive emotions rather than memory emotions (Cuddy et al., 2015). Fifth, any individuals with obvious symptoms of anxiety or depression were excluded. Sixth, participants were required to refrain from smoking or drinking alcohol, coffee, or other beverages that stimulate the sympathetic nervous system during the 6 h before the test (Li and Kang, 2019). Finally, written informed consent was obtained from all participants before the test began.

### Activity

To select the type of activity that would best facilitate the sound intervention experiment, we visited seven elderly care facilities in northern China to select older people with dementia. Through observation, we identified that older peoplewith dementia participated in painting, origami, singing, gardening, finger exercises, Tai Chi, ball sports, card games, watching TV, and walking. Finger exercises were selected as the activity for the experiment in this study for four reasons: First, of the abovementioned activities, finger exercises were the most actively participated activity by older people with dementia in the seven elderly care facilities. Second, they are convenient for capturing facial expressions because participants are seated during the exercise, facing forward, and body movements are relatively less. Third, for the collective activity of finger exercises, the error caused by the number of experiments can be reduced. Finally, the finger exercise itself does not produce noise, so it will not interfere with the sound intervention activity.

### Experiment Site

Emotion experiments are usually carried out in the field or a laboratory. Field experiments are conducted in a naturally occurring environment, with high reliability and authenticity (Harrison, 2016). A key consideration in this study is that elderly with dementia are particularly sensitive to unfamiliar environments. Thus, to ensure that the participants were as comfortable as possible and thereby to improve the reliability and validity of the results, it was necessary to implement the intervention in a place familiar to them (El Haj et al., 2015). After considering the sensitivity of participants and the collective nature of the finger exercise activity, we decided to conduct a field experiment and hence selected the public activity space of an institute in Changchun, China, as the experiment site.

### Sound Source

Some previous studies have proved that the following six types of sound sources may help to improve mood, namely, music, birdsong, fountain, stream, wind/rain, and wind/leaves (Zhongzhe, 2016; Hong et al., 2020). Birdsong was mainly concentrated in the high-frequency region and other sound sources are mainly concentrated in low frequencies, while the sound of the music had an obvious rhythm. An external speaker was used for the output of the sound source for the experiment, as prolonged use of a headset would cause the participants to become uncomfortable and would interfere with the experimental results. As it is difficult to distinguish between the emotions induced by the music and the lyrics of songs, instrumental music is more suitable for use in such an experiment (Cuddy et al., 2015). Therefore, we selected a piano performance of “Red River Valley” as the music intervention stimulus: the song was included in the Chinese Academy Award film with the same name, released in 1996. The film *Red River Valley* shows the heroic and unyielding national spirit of the Chinese people and is well-known among older adult participants. A previous study on music therapy showed that this song has the effect of regulating emotions (Shuping et al., 2019).

**Figure 1:**
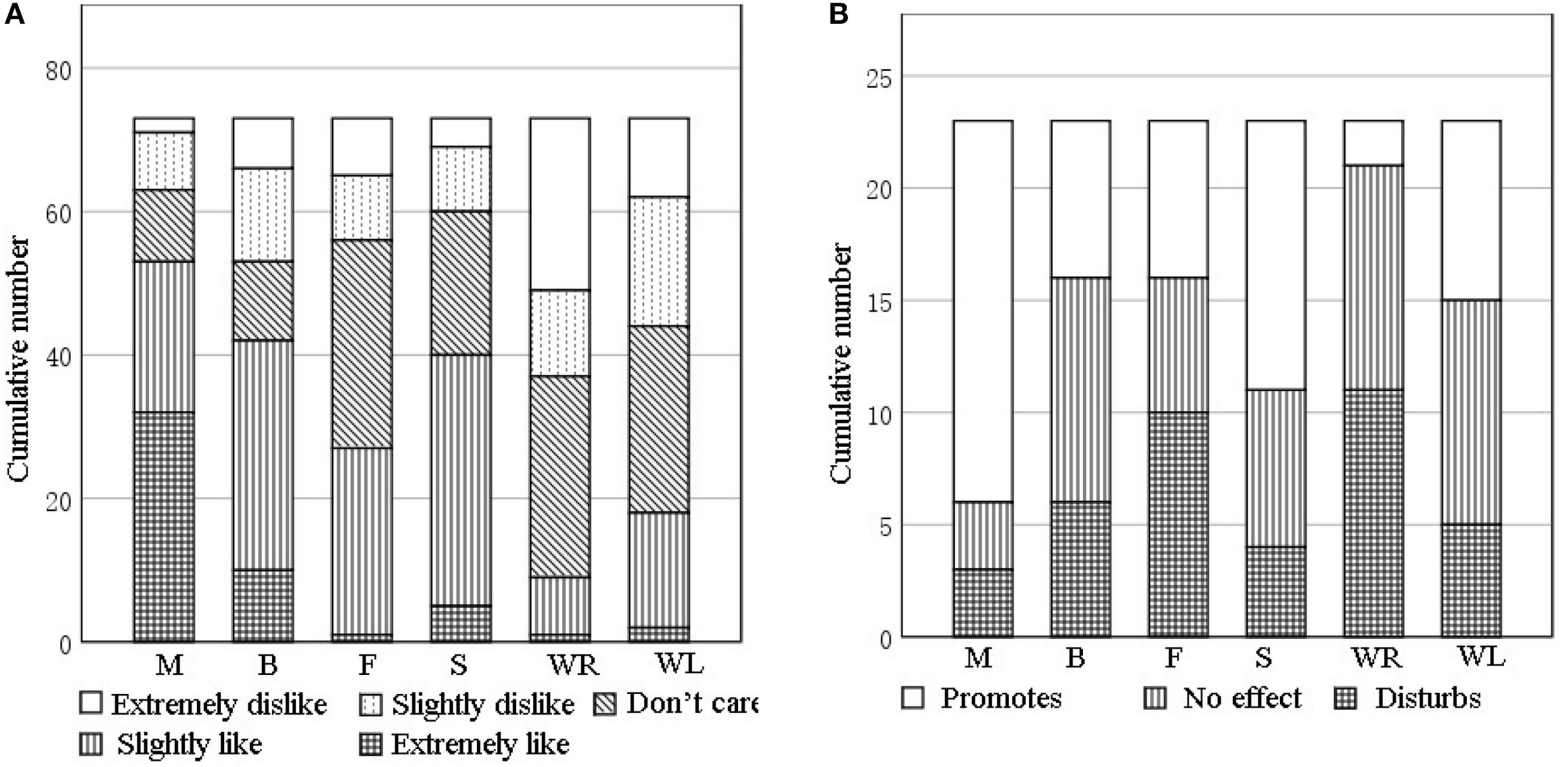
**(A)** Sound type preferences of older adults with dementia. **(B)** Evaluation of the effect of each sound type on care partners on activity engagement; M, Music; B, Birdsong; F, Fountain; S, Stream; WR, wind and rain; and WL, wind blowing leaves.

To deepen the understanding of sound source preferences by participants while also considering the impact of sound interventions on the work of care staff, a survey was conducted. Across the seven elderly care facilities, a total of 73 older people with dementia (35 men, 38 women; mean age = 79, SD = 9) were surveyed on their sound source preferences. The 1-min equivalent sound pressure level (SPL) was adjusted to 55 dB(A) for each audio frequency by AuditionCS6 to remove differences in volume during the stimulation of the four sounds and to ensure that the participants listened to the four auditory stimulus sounds under similar playback SPL conditions. The background noise was below 45 dB(A) during the survey (Zhou et al., 2020). The selected retirement facilities met the following two criteria: (1) providing sufficient daily activities and being fully equipped to ensure that the conditions in which older adults reside would not affect their evaluation of the sound sources and (2) having 10 or more residents, allowing efficient distribution of the questionnaire and increasing the statistical reliability of collected data. Each sound source was played in a loop for 1 min. At the end of one sound source, participants had 10 s to conduct a sound preference questionnaire for the sound source. We used a Likert scale in the sound preference questionnaire, as its structural simplicity and relative clarity make it particularly suitable for completion by elderly with dementia. The questionnaire design is outlined in Table 1. We also surveyed 23 care partners (mean age = 36, *SD* = 12; 6 males, 17 females) of older people with dementia to collect their insights on the extent to which each sound source affects their work. The statistics are shown in Figure 1.

**Table 1:** Contents of the sound preference questionnaire for older adults with dementia.

**Sound preference questionnaire**		**Description**
Demographic information		Gender, age
Sound source type	Music	1 = Extremely dislike
	Birdsong	2 = Slightly dislike
	Fountain	3 = Don't care
	Stream	4 = Slightly like
	Wind and rain	5 = Extremely like
	Wind blowing leaves	

A one-sample *t*-test with 3 (meaning do not care in the questionnaire) as the test value was performed on the preference scores for different types of sound sources. As shown in Table 2, elderly with dementia liked music (*p* = 0.001, *t* = 7.56), birdsong (*p* = 0.018, *t* = 2.42), and the sound of a stream (*p* = 0.001, *t* = 3.34) but disliked the sound of wind and rain (*p* = 0.001, *t* = −5.36) and of wind blowing leaves (*p* = 0.03, *t* = −2.21); their evaluation of the fountain sound was neutral (*p* = 0.724, t = 0.35). We also performed a one-sample *t*-test on the degree to which each sound source affected the work of care partners. The results show that care partners believed music (*p* = 0.001, *t* = 4.04) and the sound of a stream (*p* = 0.043, *t* = 2.15) would promote their work; the sound of wind and rain (*p* = 0.009, *t* = −2.86) would disturb the work; but birdsong (*p* = 0.788, *t* = 0.27), the sound of a fountain (*p* = 0.497, *t* = 0.72), and the sound of wind blowing leaves (*p* = 0.418, *t* = −0.83) would have no effect. Based on these findings, we selected music, birdsong, and the sound of a stream to be the sound sources for the interventions in our field experiment as sounds preferred by older adults and that will not disturb the work of care partners.

**Table 2:** Comparison of the degree of preference of older adults with dementia on different types of sound sources.

	**M**	**B**	**F**	**S**	**WR**	**WL**
Score	4.00 ± 1.13	3.34 ± 1.20	3.04 ± 0.99	3.38 ± 0.98	2.32 ± 1.09	2.73 ± 1.06
*t*-value	7.56^**^	2.42^*^	0.35	3.34^**^	−5.36^**^	−2.21^*^
*p*-value	0.001	0.018	0.724	0.001	0.001	0.030

** *p < 0.01*,

* *p < 0.05; M, music; B, birdsong; F, fountain; S, stream; WR, wind and rain; and WL, wind blowing leaves*.

**Figure 2:**
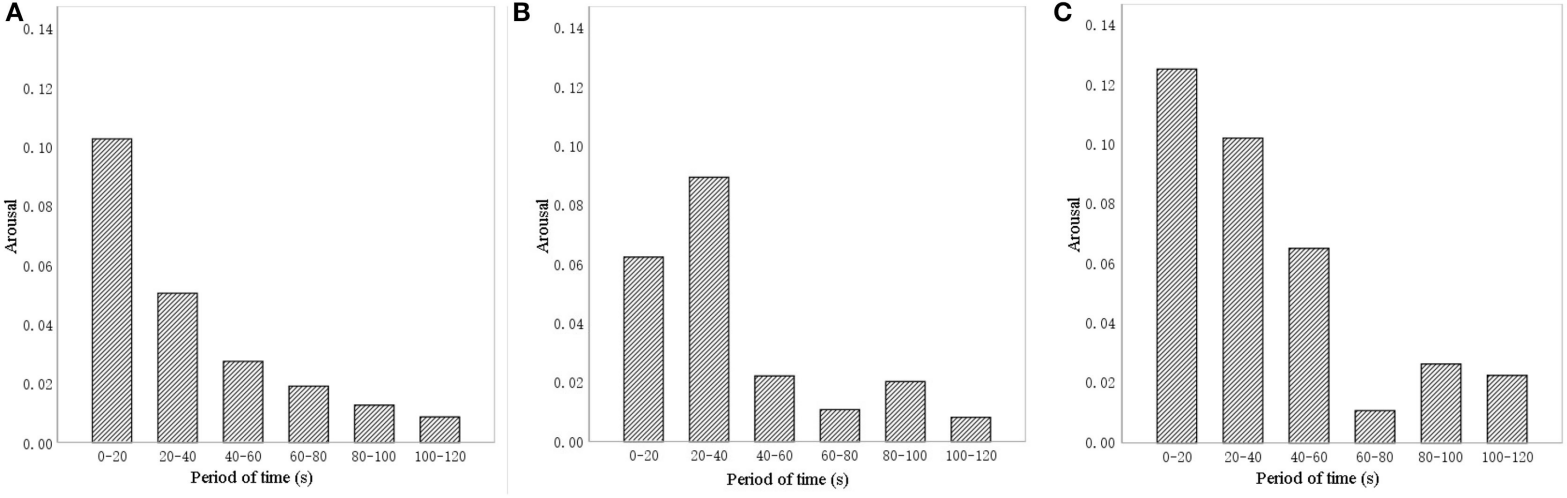
Pilot study results for arousal by the different sound interventions. **(A)** Music, **(B)** stream, and **(C)** birdsong.

To avoid any change in sound during the activity that could shape the activity effect of participants, the sound intervention time was set to match the finger exercise time (4 min 20 s), and the SPL was set to 60 dBA (El Haj et al., 2015). We recognized that, if the experiment time was too long, participants would be distracted, thus harming the accuracy of collected data. To determine a suitable analysis time, a pilot study was conducted, setting the FER sampling rate at 15/s and measuring arousal, which ranges between 0 (inactive) and 1 (active). Figure 2 shows changes of arousal in the first 120 s, with the absolute value of arousal determined every 20 s for each of the three sound sources. The trends are similar: during 0–20 s and 20–40 s, the arousal was the largest; the subsequent arousal decreased significantly, and then remained relatively stable until the end of the recording. However, in the trials with the sound of a stream and with birdsong, arousal rose again after 80 s, which may be due to distraction among the participants. The result is consistent with previous research findings (Meng et al., 2020a). Accordingly, we chose the first 80 s as the duration for analysis in our experiment.

### Emotional Evaluation Scale Design

To enable comparison with the data collected using FaceReader, we divided the subjective evaluation scale into three parts. The first part is the emotion scale, for which we selected the SAM—a nonverbal tool for self-assessment of emotions devised by Bradley and Lang (1994). The SAM can be used for people with different cognitive levels and with different cultural backgrounds, including children and adults (Ying et al., 2008; Peixia et al., 2010), and is simple and easy to operate. It includes three dimensions: arousal, pleasure, and dominance. Each dimension has five images depicting different levels, each with an associated point between the two pictures. The SAM can quickly quantify the emotional state of the subject on the three dimensions without the need for them to verbalize emotions. Backs et al. (2005) confirmed that the three dimensions of the SAM have high internal consistency. The SAM has been successfully applied in studies of people with dementia, especially those with mild-to-moderate dementia, including memory impairment. It can be used to objectively express the subjective emotional experience of dementia (Blessing et al., 2010; Lixiu and Hong, 2016). In the second part of the subjective evaluation scale, we included a question asking participants to indicate their acoustic comfort with the sound source (see Table 3). The third part of the survey collects demographics, including the age of the participant, gender, MMSE score, and other information. These data were obtained by asking the care partners or checking the medical records of the participants.

**Table 3:** Contents of the emotional evaluation scale.

**Subjective evaluation**		**Range**
Acoustic comfort		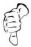 Very uncomfortable (1) to very comfortable (5) 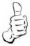
Emotion	Pleasure	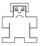 Very unpleasant (1) to very pleasant (9) 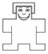
dimension	Arousal	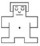 Very sleepy (1) to very excited (9) 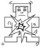
	Dominance	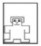 Very passive (1) to very proactive (9) 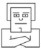

### Facial Expression Recognition

FaceReader recognizes facial expressions through a three-step process. The first step is detecting the face (Viola and Jones, 2001). The second step is accurate 3D modeling of the face using an algorithmic approach based on the Active Appearance Method (AAM) (Cootes and Taylor, 2000). In the last step, facial expressions are classified by training an artificial neural network: the AAM is used to compute scores of the probability and intensity of six facial expressions (happiness, surprise, fear, sadness, anger, and disgust) on a continuous scale from 0 (absent) to 1 (fully present) (Lewinski et al., 2014). FaceReader also calculates the valence and arousal of facial expressions. Valence refers to the emotional state of the participant, whether positive (from 0 to 1) or negative (from −1 to 0), while arousal indicates whether the test subject is active or not (from 0 to 1) (Frijda, 1986).

FaceReader inputs can be pictures or videos of a human face, and the software supports offline video input. In comparison with the pictures, videos enable more data to be generated, and the output data can be connected to reveal changes in trends over time. Therefore, we selected videos as the input in our experiment. In the video-recording process, the subject must always face the camera, and only a small angle of rotation is allowed. Older people with dementia can fully meet these requirements when performing finger exercises. The number of FaceReader online recording devices is limited. Therefore, we recorded offline videos of the facial expression of the subject.

**Figure 3:**
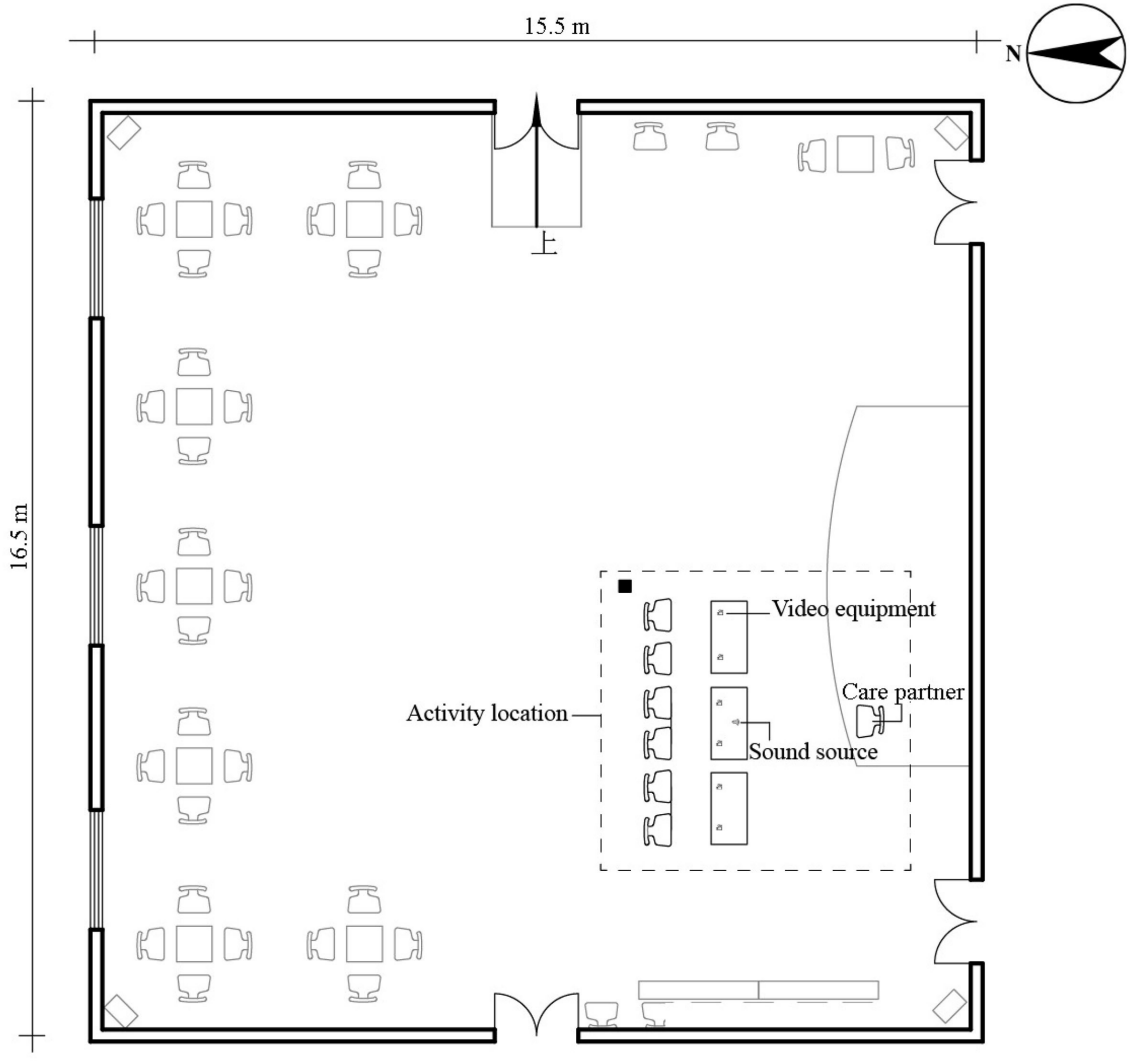
Layout of the experimental site.

The experiment site selected was the indoor public activity space of an elderly care facility in Changchun (15.5 × 16.5 × 2.8 m). Figure 3 shows the layout of the room, delineating the main experiment site within the dotted frame, where participants performed the finger exercises. The site was equipped with seven chairs, three tables, video equipment, and a sound source. The video equipment was an iPhone, placed 0.5 m from each elderly person and 0.5 −1.5 m from the sound source. Because the mobile phone can meet the video pixel requirements of FER software and its size is small, it is convenient to use it with a bracket fixed to the table, which will not make older adults fearful. The care partner was positioned at 2 m from the participant to offer guidance. Throughout the experiment, the doors and windows of the room were closed. To ensure that neither the indoor temperature nor the level of illumination affected the mood and performance of the participant (Altomonte et al., 2017; Petersen and Knudsen, 2017), we ran the experiment from 10:00 to 11:00 in the morning and maintained the temperature at 23 to 25°C (Nematchoua et al., 2019).

**Figure 4:**
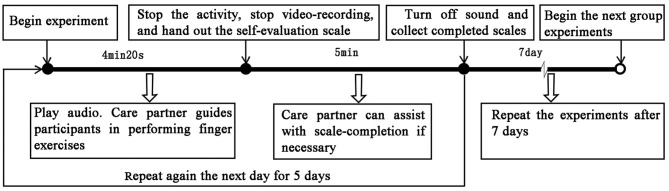
Experimental procedure steps.

In the pilot study, it was found that the intervention effect disappeared 1 week after stopping the intervention (section Data Analysis). Therefore, the sequencing effect of the stimuli can be ignored. Thirty-five participants were arranged to repeat each set of experiments. To avoid distraction by including too many people during exercise, a total of 35 participants were randomly allocated into groups of 7 for an exercise before each experiment. After the first group was seated, the designated sound source was played and the care partner guided participants in performing finger exercises for 4 min and 20 s. It is worth noting that, in no sound group experiment, the speaker was turned off. Subsequently, the emotional evaluation scale was issued for completion by the participants, with assistance from care partners where necessary, within a 5 min window. In turn, other groups undertook the same process to complete one experiment. The experiment was repeated for 5 days under the same sound source. The experiment interval of different groups was 1 week (Meilan Garcia et al., 2012). The flow of the experiment is shown in Figure 4.

### Data Analysis

Statistical Product and Service Solutions (SPSS 23.0) was used to analyze the survey data. Data with a large degree of dispersion were removed. In the pilot study, we performed an independent-samples *t*-test between valence data before and 1 week after the first intervention (before the second intervention). The pre-intervention valence (−0.173 ± 0.086) and the 1-week-post-intervention valence (−0.141 ± 0.096) did not significantly differ (*p* = 0.297). This indicates that, although the experiments were performed by the same groups of participants, the intervention effect disappeared within a week, meaning that the groups can be considered independent in each experiment. Therefore, a one-way ANOVA was used to record the differences in valence from the results of interventions with different sound sources. Linear, quadratic, and cubic regression analyses were used to analyze the changes in valence and facial-expression indicators over time. Then, the repeated measurement method was used to test the changes in potency on different days of the experiment. We also used Pearson's correlations to calculate the relationship between the results from FaceReader and the results from the emotional evaluation scale and to identify individual differences. Effect sizes were also reported using an effect size calculator, represented by the sign r (Lipsey and Wilson, 2000). TheA point-biserial correlation was used to determine the relationship between gender and test results.

## Results

### The Effects of Sound Interventions on the Facial Expressions of Older Adults With Dementia

**Figure 5:**
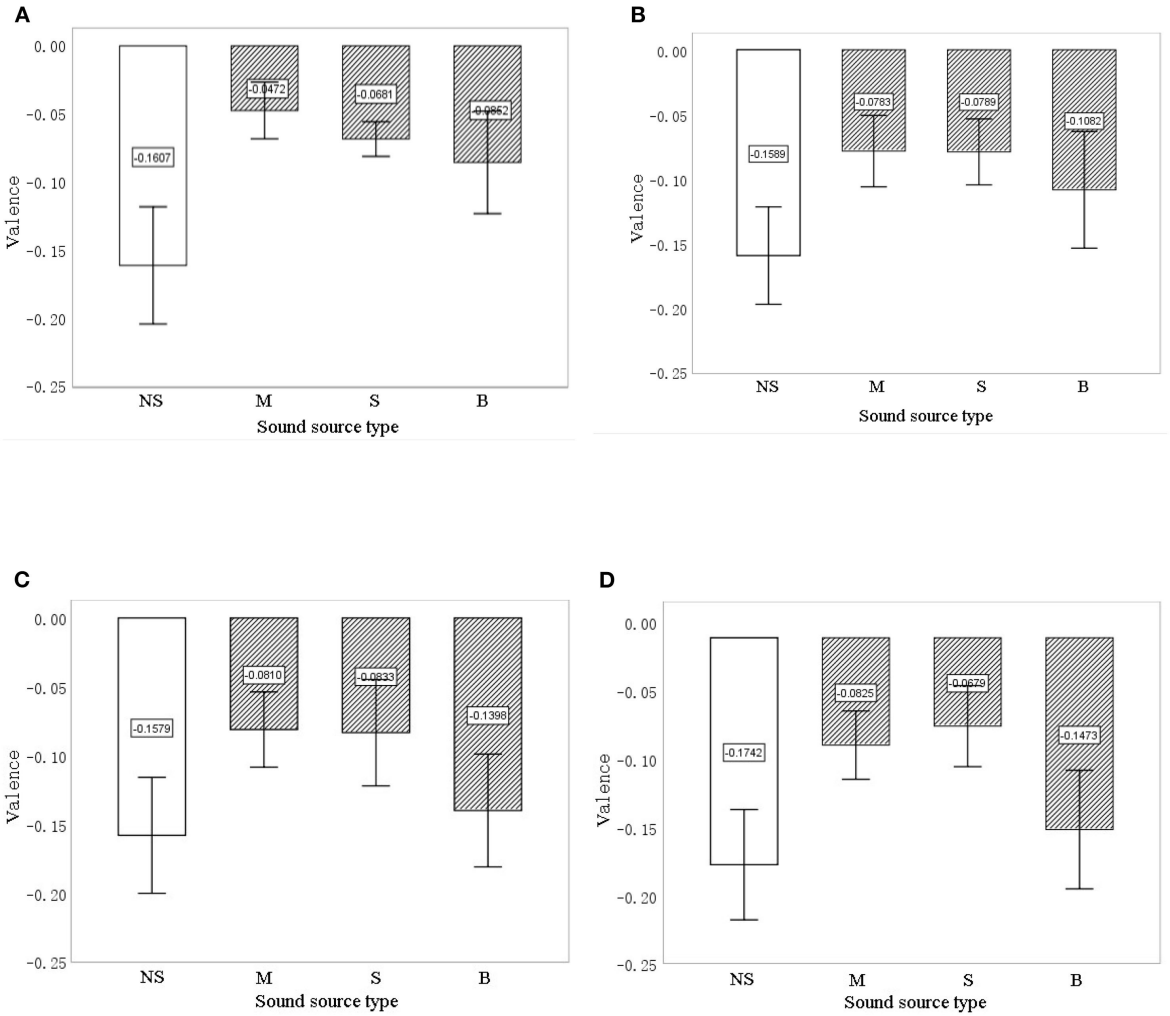
The valence for the different intervention sound types and the no-sound intervention at 20 s **(A)**, 40 s **(B)**, 60 s **(C)**, and 80 s **(D)**. The error bars show 95% CIs. NS, no sound; M, music; S, stream; and B, birdsong.

FaceReader calculates the valence for the facial expression in each frame. Each individual has different initial values for facial expressions in their natural state. Therefore, experiments with no sound intervention were performed to provide initial data. The average valence after 20, 40, 60, and 80 s with no sound intervention and with the three types of sound interventions was compared. Figure 5 shows how average valence changed from 20 to 80 s (error bars represent the 95% confidence interval). Valence was higher for the sound interventions than for the no-sound intervention. The valence of birdsong had the greatest drop (from −0.085 to −0.147), followed by music (from −0.047 to −0.083). The valence of music was always the highest at 20 s (−0.047) and 60 s (−0.081); the valence of the sound of a stream dropped from 20 s (−0.068) to 60 s (−0.083) but has the highest valence at 80 s (−0.068). The valence for the no-sound intervention increased from 20 s (−0.161) to 60 s (−0.158), then decreased again at 80 s (−0.174). To determine the difference between sound interventions, ANOVA was carried out. Significance at 20, 40, 60, and 80 s was 0.001, 0.001, 0.003, and 0.001, respectively; this indicates that, after intervention for 20, 40, 60, and 80 s, the type of sound source had a significant effect on the facial expressions of elderly with dementia.

To test the difference between various sound source types, a multiple comparison analysis was also carried out. As Table 4 shows, the biggest difference in valence was between no sound and music sound, with an average difference of 0.769 at 60 s (*p* = 0.001), followed by the difference between no sound and stream, with an average difference of 0.746 at 60 s (*p* = 0.008). The average difference in valence between birdsong and no sound was significant at 20 s (*p* = 0.049) and 40 s (*p* = 0.038), but non-significant at 60 s and 80 s. In addition, valence was mostly similar between music and stream. However, valence differed significantly between music and birdsong at 60 s (average difference = 0.059, *p* = 0.025) and between stream and birdsong at 80 s (average difference = 0.794, *p* = 0.029). The valence results in Figure 5 and Table 4 show that the interventions with sound sources have a positive effect on the valence of facial expressions of older adults with dementia compared with the no-sound source interventions. In addition, there are differences in valence between the sound source types at different time points in the intervention, which indicates that FaceReader can identify differences in the emotional responses of older people with dementia to different intervention sound sources.

**Table 4:** The average difference in valence between different sound source types at 20, 40, 60, and 80 s during the intervention.

**Intervention time point**	**NS&M**	**NS&S**	**NS&B**	**M&S**	**M&B**	**S&B**
20 s	−0.113^**^	−0.096^**^	−0.075^*^	0.021	0.017	0.170
40 s	−0.081^**^	−0.080^**^	−0.051^*^	0.001	0.038	0.029
60 s	−0.769^**^	−0.746^**^	−0.018	0.022	0.059^*^	0.057
80 s	−0.092^**^	−0.106^**^	−0.289	-0.145	0.648	0.794^*^

** *p < 0.01*,

* *p < 0.05; NS, no sound; M, music; S, stream; and B, birdsong*.

**Figure 6:**
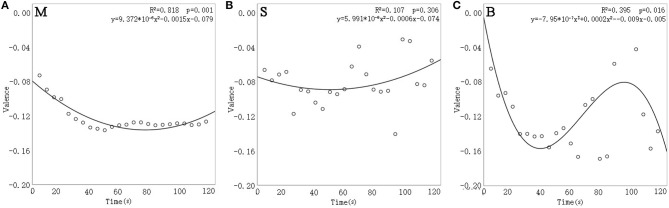
The relationship between valence and time for the sound interventions of music **(A)**, stream **(B)**, and birdsong **(C)**.

By performing linear, quadratic, or tertiary regression analysis on each intervention, Figure 6 shows the trend of valence over time for each of the three sound interventions. Valence changed significantly over time for music (*p* = 0.001) and birdsong (*p* = 0.016) sound interventions but the valence for the sound of a stream intervention did not change. In the music intervention, valence decreased at around 60 s by 0.058 and then recovered slightly. In the birdsong intervention, valence dropped by 0.091 from 0 to 40 s, then rose by 0.138 until 100 s, before subsequently declining again. These results demonstrate that FaceReader can reflect how the facial expressions of elderly with dementia change over time.

### The Influence of Sound Interventions on Facial Expression Indices

**Figure 7:**
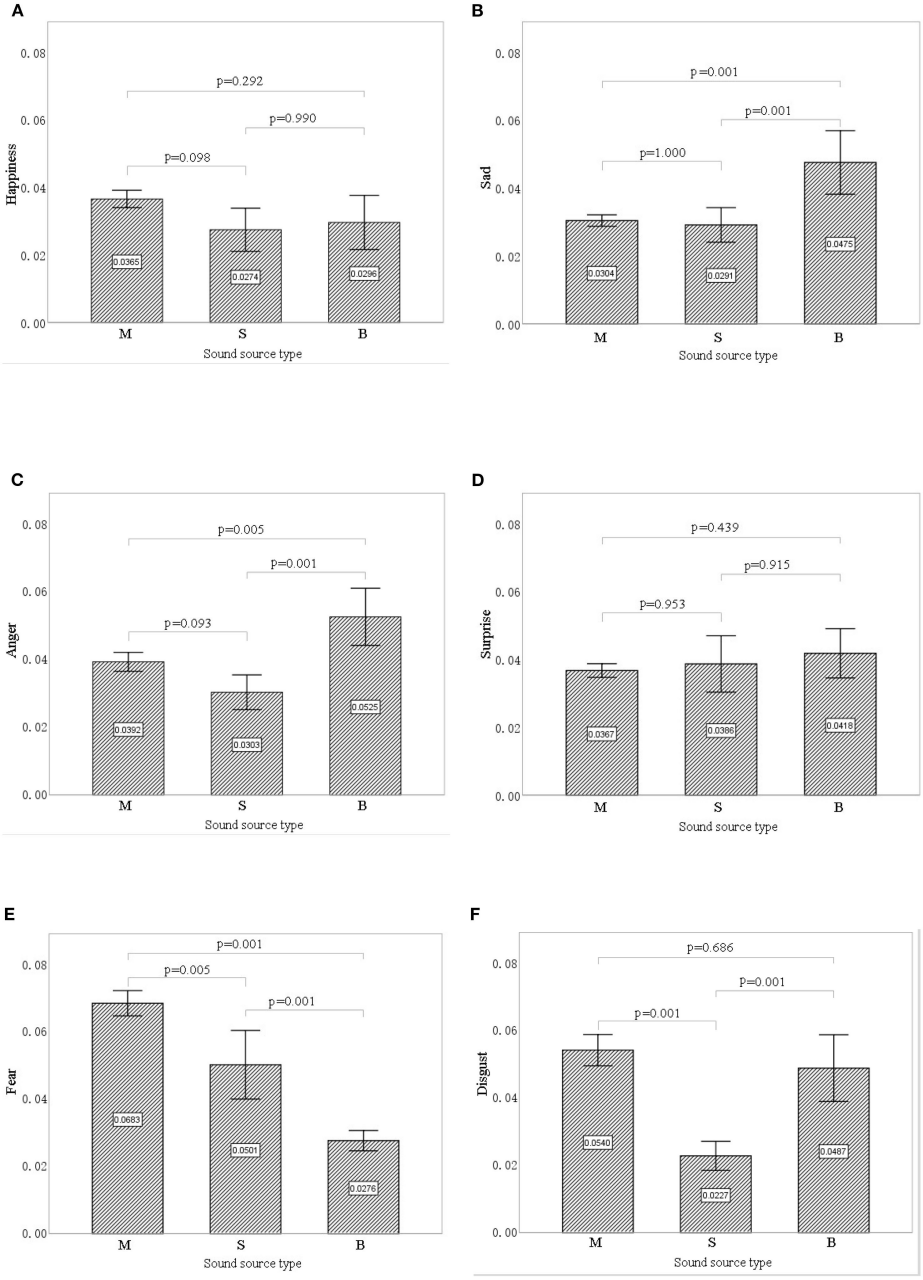
The effect of each sound source type on different facial expression indices: **(A)** happiness, **(B)** sadness, **(C)** anger, **(D)** surprise, **(E)** fear, **(F)** disgust. Error bars show 95% CIs. M, music; S, stream; B, birdsong.

Figure 7 shows the differences in facial expression indices of the participants between the three types of sound sources. Sadness (mean = 0.036, *SD* = 0.015), fear (mean = 0.049, *SD* = 0.022), and disgust (mean = 0.042, *SD* = 0.021) all differed significantly between interventions (*p* < 0.01), whereas happiness (*p* = 0.081), surprise (*p* = 0.503), and anger (*p* = 0.071) did not. Therefore, facial expression indices of sadness, fear, and disgust were selected to analyze the impacts of different sound interventions.

**Figure 8:**
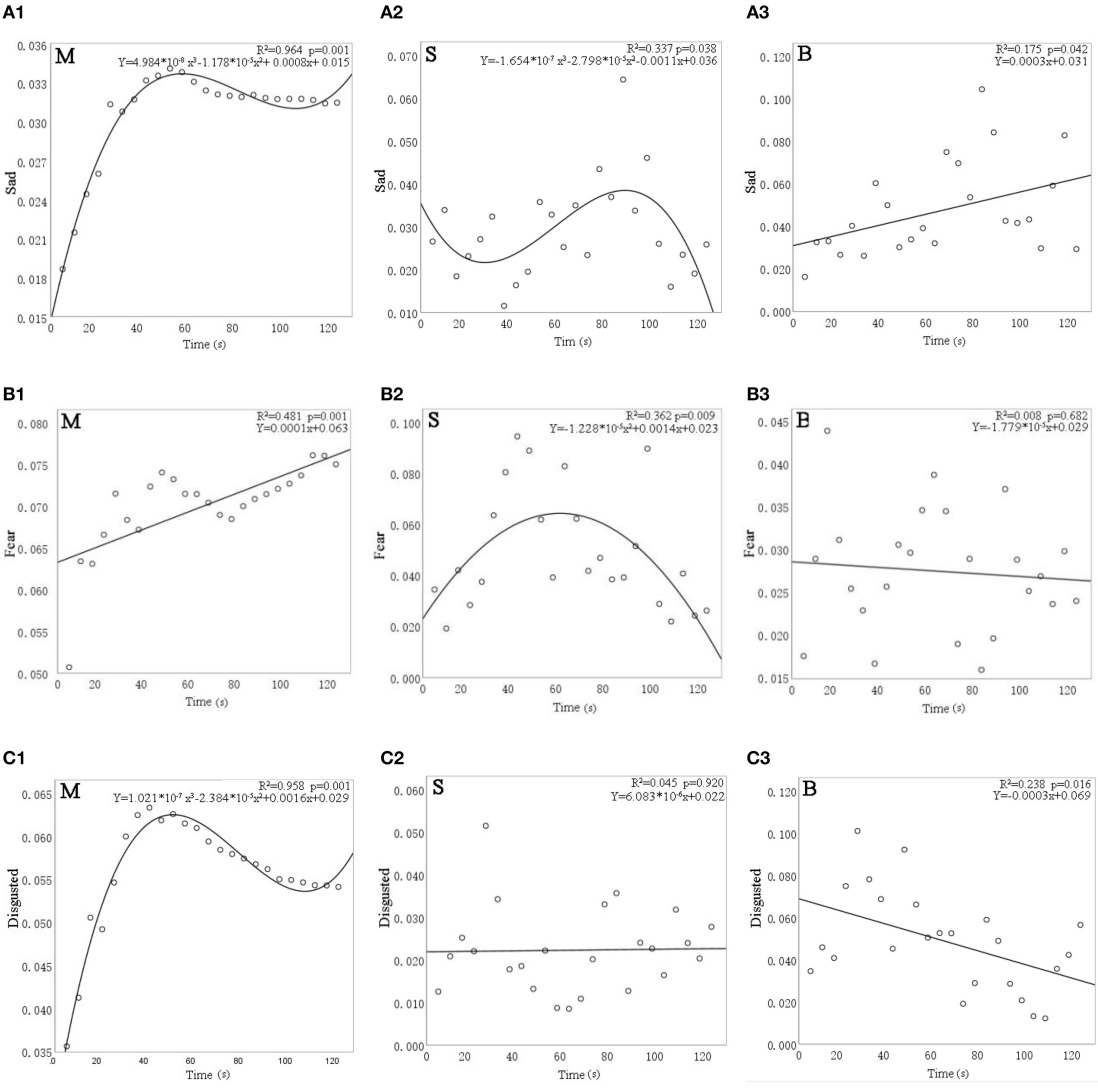
The relationship between facial expression indices—**(A)** sadness, **(B)** fear, and **(C)** disgust—and time for different sound interventions. M, music; S, stream; and B, birdsong.

Figure 8 shows the results of linear, quadratic, and cubic regression analyses for the facial expression indices of sadness, fear, and disgust. All three expression indices were significantly affected by time except for disgust with the sound of a stream (*p* = 0.920) and for fear with the birdsong intervention (*p* = 0.682).

Focusing first on sadness, Figure 8A shows that, for the music intervention, sadness expression increased by 0.015 from 0 to 40 s before gradually decreasing. For the stream sound, sadness dropped by 0.003 from 0 to 20 s and then gradually rose by 0.014 until 80 s, before subsequently decreasing again. For birdsong, sadness gradually increased over time (by ~0.01 every 20 s).

Turning to fear, Figure 8B shows a gradual rise from 0 to 50 s (0.009 every 20 s) for the music intervention and then a decrease of 0.007 from 50 to 80 s, followed by a linear rise (of 0.002 every 20 s). For the stream sound, fear expression increased by 0.021 in the first 60 s and then decreased by 0.035 from 60 to 120 s. For birdsong, the fear expression did not change significantly over time (*p* = 0.682).

Regarding disgust, Figure 8C shows a rapid rise of 0.028 from 0 to 40 s for the music intervention and then a slow drop of 0.008 from 40 to 100 s. For birdsong, disgust increased by 0.06 from 0 s to about 20 s and then showed a linear downward trend, with an average decrease of 0.03 every 20 s. For the stream sound, disgust expression did not change significantly over time (*p* = 0.920).

The above results show that, under different sound interventions, sadness, fear, and disgust are all significantly affected by time. Therefore, in the study of emotions in older adults with dementia, these facial expression indices can be used to evaluate the effects of emotional intervention.

### The Influence of Time on Facial Expressions of Elderly With Dementia

To explore the influence of intervention duration on facial expressions, we conducted a further set of experiments in which the intervention sound (music) began to be played 2 min before the exercise started (advance group) and at the beginning of the exercise (normal group). The other experimental steps were unchanged.

**Figure 9:**
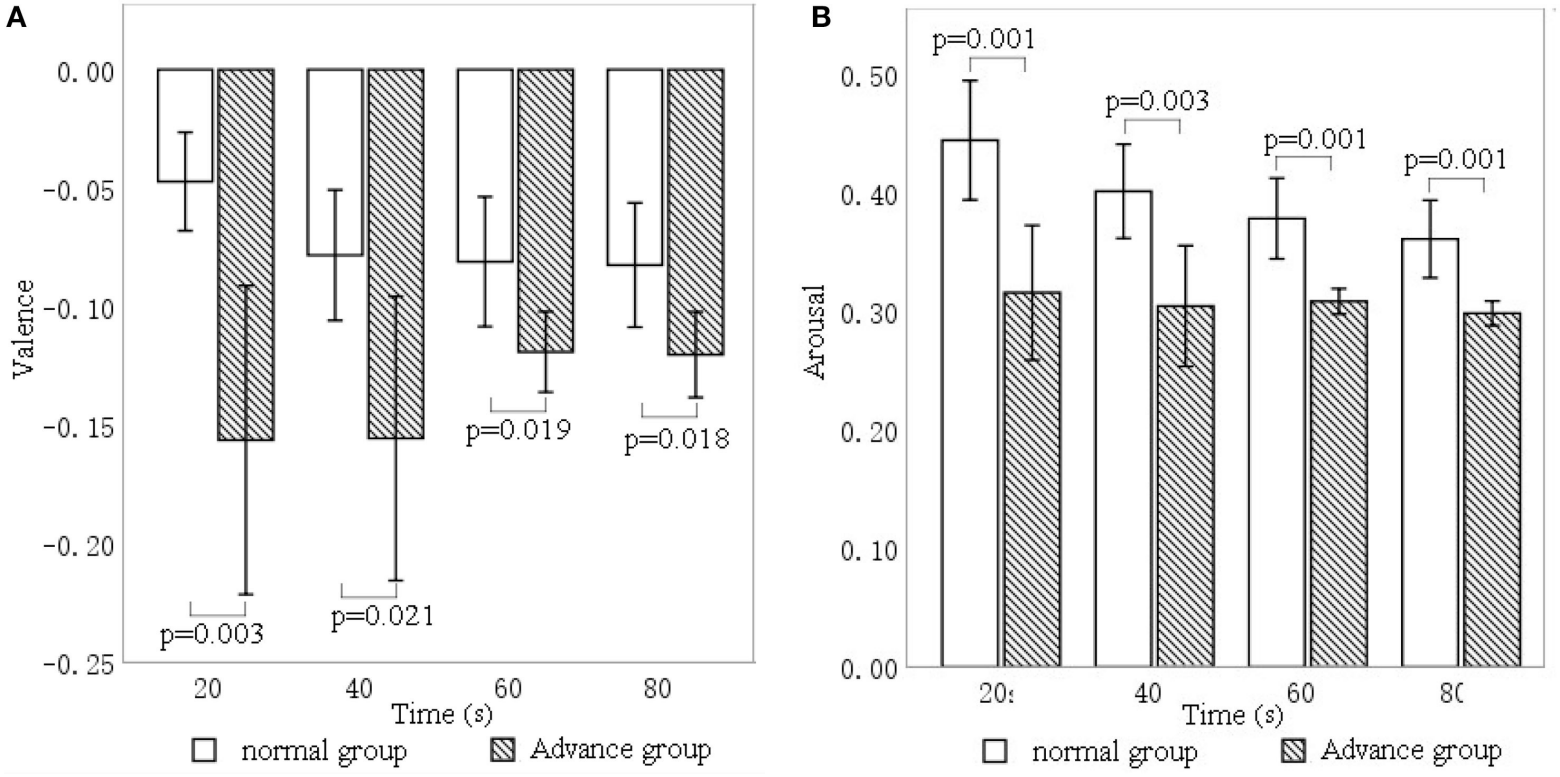
Differences in **(A)** valence and **(B)** arousal for music intervention of different durations.

Figure 9A shows that valence significantly differed between the advance group and the normal group at 20 s (*p* = 0.003), 40 s (*p* = 0.021), 60 s (*p* = 0.019), and 80 s (*p* = 0.019). In addition, valence in the advance group (mean = −0.156, −0.156, −0.119, −0.120) was significantly lower than that in the normal group (mean = −0.047, −0.07, −0.081, −0.082) at the four respective time points.

In terms of arousal, Figure 9B shows significant differences between the advance group and the normal group at 20 s (*p* = 0.001), 40 s (*p* = 0.003), 60 s (*p* = 0.001), and 80 s (*p* = 0.001). Moreover, arousal in the advance group (mean = 0.315, 0.304, 0.308, 0.298) was significantly lower than in the normal group (mean = 0.444, 0.401, 0.378, 0.361) at the four respective time points.

### The Influence of Intervention Duration on Facial Expression Indices

**Figure 10:**
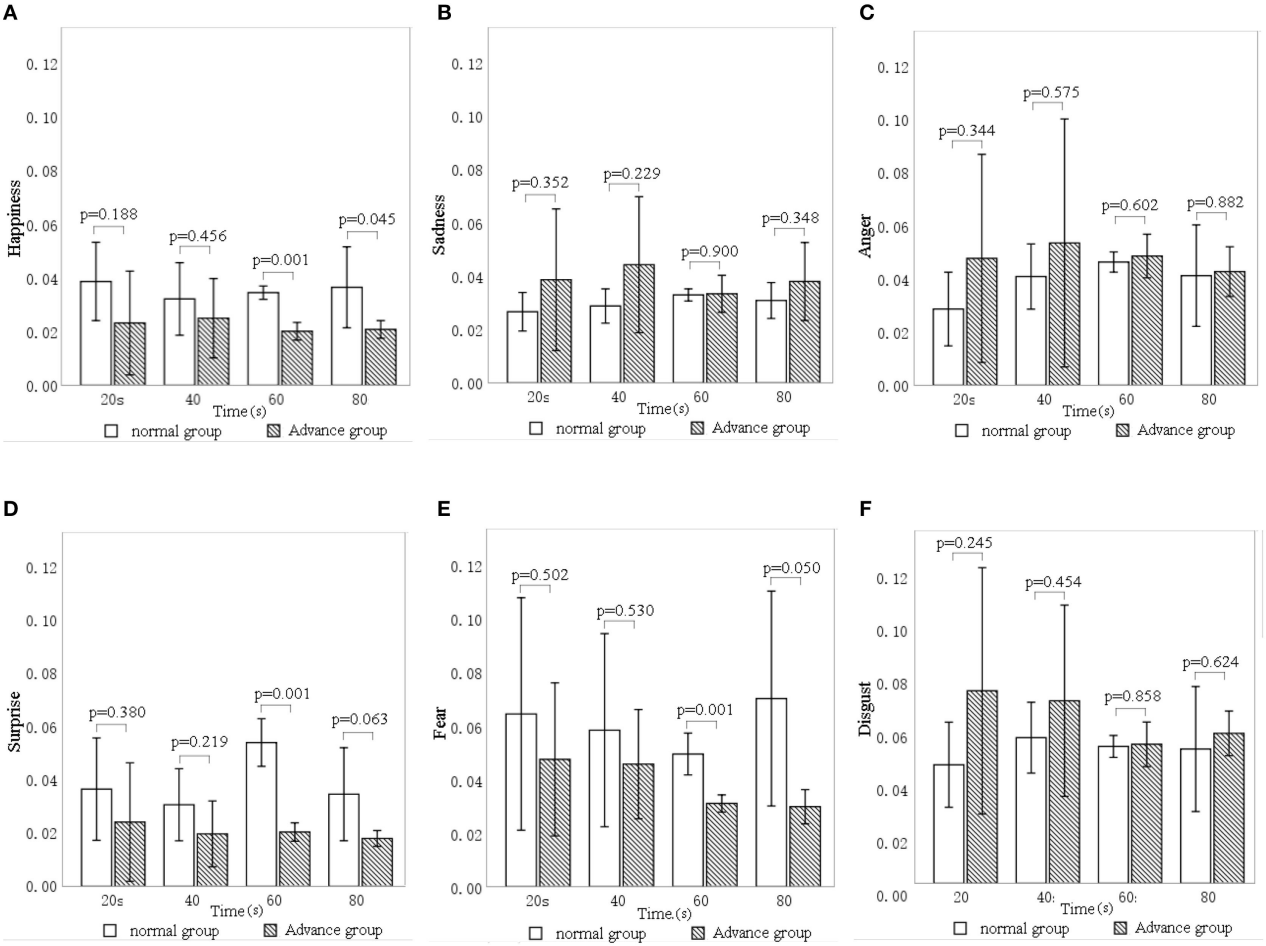
Differences in facial expression indices—**(A)** happiness, **(B)** sadness, **(C)** anger, **(D)** surprise, **(E)** fear, **(F)** disgust—for different intervention durations.

Figure 10 shows the relationship between the intervention duration (advance group and normal group) and the six facial expression indices. There are significant differences between the advance and normal groups in happiness (*p* = 0.002), fear (*p* = 0.018), and surprise (*p* = 0.001).

Figure 10A shows that happiness expression was significantly lower in the advance group (mean = 0.022, SD = 0.024) than in the normal group (mean = 0.035, *SD* = 0.026), with the largest difference at 80 s. Figure 10D shows that surprise expression was also significantly lower in the advance group (mean = 0.021, *SD* = 0.026) than in the normal group (mean = 0.039, *SD* = 0.032), with the largest difference at 60 s. Figure 10E shows that fear expression in the advance group (mean = 0.038, *SD* = 0.036) was significantly lower than that in the normal group (mean = 0.060, *SD* = 0.071), most substantially at 80 s.

## Discussion

To decide whether and how FaceReader can take the place of questionnaires as a tool in sound perception research, the results of these two methods should be discussed. As mentioned earlier, FaceReader can recognize the facial expressions of older people with dementia. Whether FaceReader can replace the subjective evaluation scale as a tool for emotional research in older people with dementia is discussed. Bivariate Pearson correlations were used to analyze the relationship between the subjective emotional evaluation of the participant and facial expression valence, reporting the effect size of Cohen'*d* (Table 5). Based on the sign of r, the valence of facial expressions is positively correlated with the subjective evaluation of pleasure, arousal, superiority, and acoustic comfort. In the music intervention, pleasure (r ranging from 0460 to 0.679) is significantly correlated with valence at all four-time points. Dominance (r ranging from 0.282 to 0.600) and acoustic comfort (r ranging from 0.202 to 0.545) were significantly correlated with valence at 60 s and 80 s, while arousal (*r* = 0.468) was significantly correlated with valence at 20 s. For the sound of a stream, valence change in the first 60 s can be used to predict arousal (r ranging from 0.061 to 0.866), pleasure (r ranging from 0.021 to 0.0762), and acoustic comfort (r ranging from 0.102 to 0.760), while dominance can be reflected by valence change at 20 s (*r* = 0.790). In the birdsong intervention, valence change in the first 60 s can be used to predict pleasure (*r* = 0.830), arousal (*r* = 0.891), and acoustic comfort (r ranging from 0.769 to 0.907), while dominance (*r* = 0.824) can be represented by valence at 20 s.

**Table 5:** The relationship between subjective emotional evaluation and facial expression valence in three sound interventions at 20, 40, 60, and 80.

**Sound source type**		**Subjective emotional evaluation**			
	**Time**	**Pleasure**	**Arousal**	**Dominance**	**Acoustic comfort**
Music	20 s	0.601^**^	0.468^*^	0.282	0.202
	40 s	0.679^**^	0.406	0.431	0.374
	60 s	0.565^*^	0.354	0.535^*^	0.475^*^
	80 s	0.460^*^	0.343	0.600^**^	0.545^*^
Stream sound	20 s	0.021	0.061	0.790^*^	0.102
	40 s	0.770^*^	0.749	0.236	0.766^*^
	60 s	0.762^*^	0.772^*^	0.192	0.760^*^
	80 s	0.692	0.866^*^	0.064	0.697
Birdsong	20 s	0.727	0.891^*^	0.824^*^	0.769
	40 s	0.768	0.760	0.588	0.862^*^
	60 s	0.830^*^	0.844^*^	0.651	0.860^*^
	80 s	0.796	0.514	0.400	0.907^*^

** *p < 0.01*,

* *p < 0.01*.

In terms of individual differences, first, the point-biserial correlation analysis revealed that gender and facial expression are not significantly correlated in the music and birdsong interventions. This is consistent with previous research conclusions reached from evaluating acoustic environment using questionnaires (Meng et al., 2020a). However, a significant correlation was found between facial expressions and gender at 20 s in the stream sound intervention, with valence significantly higher among women than men (*r* = 0.869, *p* = 0.011). This suggests that the sound of a stream can more easily elevate the emotions of women. Regarding age, the results of the bivariate Pearson correlation analysis show a negative correlation between age and facial expression valence (*r* = −0.467, *p* = 0.044) at 80 s for the music intervention but a positive correlation at 20 s for the stream sound (*r* = −0.756, *p* = 0.049). However, there was no correlation between age and valence for the birdsong intervention. Finally, we found no correlation between the MMES score and facial expression valence for any of the three sound types.

**Figure 11:**
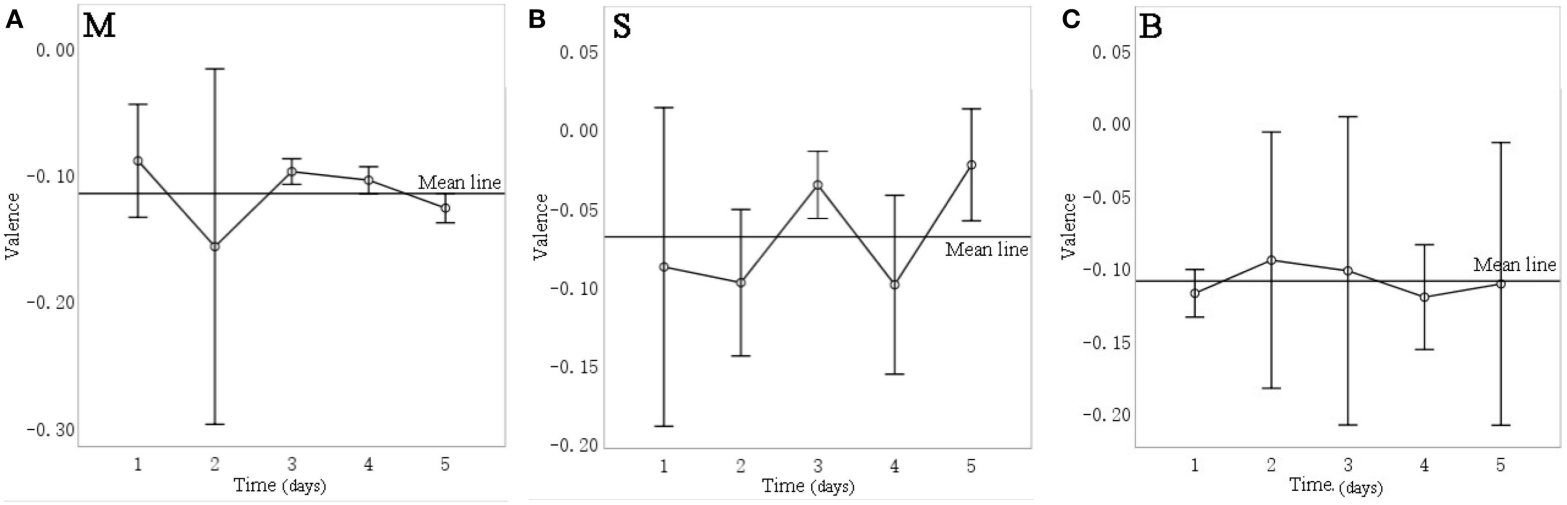
Changes in valence over 5 days of experiment for different sound interventions. **(A)** music, **(B)** stream, **(C)** birdsong.

In terms of intervention days, Figure 11 shows the mean valence of each day for different sound sources for over 5 days of intervention. Repeated measurement variance analysis reveals that the valence for music intervention on the third day (mean = −0.098, *SD* = 0.014) and fourth day (mean = −0.104, *SD* = 0.015) was significantly higher (*p* = 0.007) than that on the fifth day (mean = −0.126, *SD* = 0.016). For the stream sound, valence on the fourth day (mean = −0.099, *SD* = 0.046) was significantly lower (*p* = 0.041) than that on the third day (mean = −0.356, *SD* = 0.017) and the fifth day (mean = −0.023, *SD* = 0.029). For birdsong, however, there were no significant differences in valence between the days (*p* = 0.094). The above results indicate that the facial expressions of elderly with dementia are affected by the number of intervention days for two of the three sound sources. Therefore, in studying how sound interventions affect the mood of older people with dementia, the number of days of the intervention should be considered.

In a field experiment studying the emotions of elderly with dementia, various factors may affect the facial expressions of the participant, such as vision, smell, and the mood of the care partner. This makes it somewhat difficult to recognize emotions through only facial expressions. Acknowledging this limitation, the aim of this research was to verify the effectiveness of FER in emotion recognition for older people with dementia.

## Conclusions

This study proposes FaceReader as a potential method for evaluating the impact of sound interventions on emotions in older people with dementia. Through field experiments with 35 participants, the following conclusions were drawn.

First, FaceReader can identify differences in the emotional responses of older people with dementia using different types of sound interventions. Among the three sound sources, music showed the most positive effects on the mood of older adults with dementia. The effects of music, birdsong, and the sound of a stream were higher than that with no sound source. The facial expression indices of sadness, fear, and disgust also differed significantly between sound sources, while happiness, surprise, and anger did not.

Second, the sound and activity started simultaneously had a more positive influence on the mood of older adults with dementia than when playing the sound before the activity started, especially under the intervention of music and streams. Regarding intervention days, only music and stream sound showed significant differences in the effect between different dates. Birdsong also had differences in effect, but those differences were not significant. This shows that, when using FaceReader to measure the impact of sound interventions on emotions in elderly with dementia, more than one intervention must be performed to obtain accurate and reliable results.

The comparison of results from FaceReader and the subjective evaluation scale shows that facial expression valence can predict pleasure, arousal, dominance, and acoustic comfort.

In terms of gender, the sound of a stream more easily elevated the emotions in women than in men. In terms of age, only under the intervention of music and stream sound was age related to the emotions of older adults with dementia. Regardless of the sound source, no correlations were found between facial expression valence and MMSE scores.

## Data Availability Statement

The original contributions presented in the study are included in the article/supplementary material, further inquiries can be directed to the corresponding author/s.

## Ethics Statement

Ethical review and approval was not required for the study on human participants in accordance with the local legislation and institutional requirements. The patients/participants provided their written informed consent to participate in this study.

## Author Contributions

YL: conceptualization, validation, writing—review and editing, supervision, and funding acquisition. GY: methodology and formal analysis. ZW: investigation, data curation, and writing—original draft preparation. All authors have read and agreed to the published version of the manuscript.

## Conflict of Interest

The authors declare that the research was conducted in the absence of any commercial or financial relationships that could be construed as a potential conflict of interest.

## Publisher's Note

All claims expressed in this article are solely those of the authors and do not necessarily represent those of their affiliated organizations, or those of the publisher, the editors and the reviewers. Any product that may be evaluated in this article, or claim that may be made by its manufacturer, is not guaranteed or endorsed by the publisher.
